# Development and validation of the Male Rape Myth Acceptance Scale (MRMAS)

**DOI:** 10.1016/j.heliyon.2021.e07421

**Published:** 2021-06-27

**Authors:** Benjamin A. Hine, Anthony D. Murphy, Jamie S. Churchyard

**Affiliations:** a University of West London, UK; b University of Birmingham, UK

**Keywords:** Rape, Male rape, Rape myth acceptance, Validation, Scale

## Abstract

Despite growing recognition of male-on-male rape and its related myths, research in this area has been held back by the lack of a reliable and comprehensive measure or scale. The present work utilises a large and diverse participant sample over two studies (Study 1 N = 510, Study 2 N = 527) to validate a new Male Rape Myth Acceptance Scale (MRMAS), measuring myths falling under six principle themes: masculinity, sexuality, pleasure, perpetrators, context, and effect. Analysis suggested a two-factor scale, with ‘Blame’ and ‘Minimisation/Exoneration’ sub-scales. Both the overall scale and sub-scales demonstrate excellent reliability and construct validity, and are thus proposed as tools to enable the proliferation of future research on male rape myth acceptance, both in general and specialist populations, in an attempt to improve the experiences of male rape victims.

## 1 Introduction

Research on the nature, function, and influence of rape myths has typically focussed on incidences involving male perpetrators and female victims. Indeed, the term ‘rape myths’ is used so ubiquitously in reference to this type of violence, that definitions have required no gendered specification. For example, Bohner et al. (2009) describes so-called ‘traditional’ rape myths as ‘descriptive or prescriptive beliefs about rape (i.e., about its causes, context, consequences, perpetrators, victims, and their interaction) that serve to deny, downplay or justify sexual violence that *men commit against women*’. However, following the legal recognition of male rape in the UK in 1994 (see Jamel et al., 2008, for review), research on the rape of men by other men, including the existence of ‘male rape myths’ has increased. Such research has led to the identification of several genres of myths specific to male rape (e.g., those surrounding compromised masculinity), and the application of some ‘traditional’ rape myths to male victims (e.g., increased victim responsibility through alcohol and/or drug consumption). However, at present, no reliable, comprehensive measure of male rape myths exists, despite calls from Struckman-Johnson and Struckman-Johnson over 25 years ago ‘to develop a “male rape myth scale”’ (1994, p. 98). This study presents the first, comprehensive ‘Male Rape Myth Acceptance Scale (MRMAS)’ for the accurate measurement of male-specific rape myths.

### 1.1 ‘Traditional’ rape myths and their measurement

Since Martha Burt's pioneering paper in 1980, research on rape myths has proliferated (Burt, 1980). As described above, rape myths are defined as beliefs about rape, which serve to minimise men's sexual violence towards women in a variety of ways. This is mirrored in the definition provided by the UK Crown Prosecution Service (CPS), that a rape myth is ‘a commonly held belief, idea or explanation that is not true’, further stating that: ‘They attempt to explain events, like rape and abuse, in ways that fit with our preconceived ideas about the world – they arise from and reinforce our prejudices and stereotypes’ (CPS, 2015, p. 1). Examples of rape myths include specific beliefs regarding victims (e.g., if a woman wears revealing clothing she is partly responsible for her victimization) and perpetrators (e.g., once men reach a certain level of sexual arousal, they are unable to control their actions), as well as broad ideas about rape as a crime, such as the ‘real rape stereotype’ (i.e., the belief that legitimate rape cases occur suddenly, at night, by an aggressive stranger, with a weapon, and typically involve visible victim resistance and emotional trauma for the victim; Estrich, 1987; Horvath and Brown, 2013).

Despite some definitional conflict (Payne et al., 1999), it is widely accepted that there are four types of rape myth, those which: attribute blame to the victim for their rape (e.g., that women who dress scantily provoke rape), minimise the seriousness of rape itself (e.g., the suggestion that many claims of rape are false), remove blame from the perpetrator (e.g., the implication that men cannot control their sex drive, especially when already aroused), and suggest that rape only happens to particular kinds of women (e.g., only promiscuous women get raped; Bohner et al., 2009). Rape myths can therefore be characterised as a general cognitive schema that enables negative attributions to be made about the crime of rape and those involved (Grubb and Turner, 2012). As such, rape myths serve several important psychological functions that enable individuals to understand and make sense of negative events in their social world, maintain cognitive consistency, avoid the experience of negative affect, and rationalise problematic behaviour (Bohner et al., 2009). Rape myth acceptance then is characterised as the extent to which a person adheres to such beliefs, and research demonstrates that rape myths are held by people of all sexes, all ages and across races (Burt, 1980; Johnson et al., 1997; McGee, O'Higgins, Garavan and Conroy, 2011; Suarez and Gadalla, 2010), and are held by both victims (Peterson and Muehlenhard, 2004) and perpetrators (Marshall and Hambley, 1996).

Importantly, studies demonstrate that rape myths are not only held by the general public (Sussenbach and Bohner, 2011), but by various specialist populations both outside of and within the criminal justice system, such as counsellors (Feild, 1978), police officers (Murphy and Hine, 2019; Parratt and Pina, 2017), lawyers and barristers (Temkin, 2000; Temkin and Krahé, 2008), judges (Temkin and Krahé, 2008) and jurors (see Dinos et al., 2015, for review). Studies have also highlighted the impact rape myths have on the attribution of blame to victims and perpetrators, again both in the general population (Grubb & Harrower, 2008, 2009; Grubb and Turner, 2012), and in specialist populations such as police officers (Hine & Murphy, 2017, 2019; Sleath and Bull, 2017), as well as their influence on case investigation and progression (Hohl and Stanko, 2015; Sleath and Bull, 2017).

Such valuable research has been enabled by the existence of several reliable measures of rape myth acceptance, developed and refined over nearly 40 years. Indeed, measures including the Rape Myth Acceptance Scale (RMAS; Burt, 1980), the Attitudes Towards Rape Scale (ATR; Feild, 1978), the Illinois Rape Myth Acceptance Scale (IRMAS; Payne et al., 1999), and the Acceptance of Modern Myths about Sexual Aggression Scale (AMMSA; Gerger et al., 2007), have all provided the tools with which to accurately assess and compare myth acceptance across groups, as well as the predictors and outcomes of rape myth acceptive attitudes. Such findings have proved useful in the development of education and training programmes which address rape myths, such as those delivered to police officers in attempts to improve the experiences of victim-survivors, and to challenge societally embedded beliefs which enable ‘rape culture’ (Burt, 1980).

### 1.2 Male rape myths

It is estimated that similar research on myths relating to male rape, and the measurement of male-specific myths, is approximately 20 years behind that of research on female rape (Davies and Rogers, 2006). Nonetheless, some research detailing male myths is available, with beliefs categorised under six central themes; masculinity, sexuality, pleasure, perpetrators, context, and effect, each of which are explored in detail below. It is important to note that so-called ‘male rape myths’ operate similarly to ‘female rape myths’, despite differing in content; in that, all of the myths outlined below seek (a) to blame the victim, (b) exonerate the perpetrator, (c) minimise the severity of the incident, and/or (d) suggest that only certain groups or types of men are raped.

Unsurprisingly, male rape victims appear to be evaluated against stereotypic conceptualisations of masculinity (e.g., hegemonic masculinity as described by Connell, 2002), in a process similar to comparisons made of female rape victims against expectations associated with femininity (Campbell, 2005). For example, as female victims are punished for displays of promiscuous behaviour antecedent to their assault which violate virtuous/reputational ideals, men are criticised for behaviour which contradicts masculine ideals relating to strength (e.g., failing to fight off an attacker; Groth and Burgess, 1980). Beliefs that ‘real’ men would not allow themselves to be raped (McMullen, 1990) or that ‘real’ men *cannot* be raped (Hillman, O'Mara, Tomlinson and Harris, 1991) are also borne from such comparisons. This occurs despite evidence suggesting that men, similarly to women, engage in “passive submission, engendered by an overwhelming sense of disbelief” when attacked (King, 1992, p. 3). Moreover, men suffer additional judgment and associated shame for even becoming victims in the first place, as this again contradicts stereotypes of men as powerful, in control, dominating, and strong (Connell, 2002), and thus able to defend themselves from attack (Gonsiorek, 1994; Struckman-Johnson, 1991). Such judgments support erroneous beliefs that male victims of rape must therefore be children, or very weak adults (Scarce, 1997), and that if a man does not fight off an attacker, they must have wanted to have been ‘raped’ (Struckman-Johnson and Struckman-Johnson, 1992). Crucially, such myths predicate the idea that victimisation should and does result in a loss of, and compromise to, masculinity, along with a loss of status as a ‘real man’ (Groth and Burgess, 1980). Moreover, men who have been raped are thought to be weak and vulnerable (Isely, 1991), responsible for the assault (Hickson et al., 1994), and to blame for their victimisation (Myers, 1989).

Masculinity myths are strongly related to myths surrounding sexuality. Indeed, as it has been highlighted that traditional, restrictive and regressive masculine ideals often fuel prejudice and discrimination towards sexual minority men (E. Anderson, 2009), as they are evaluated as contradictory to those ideals. It is therefore unsurprising that homophobic assumptions about sexuality are thus made upon victimisation, and in such ways as to discredit and negatively judge the male victim, as masculine perceptions around power, dominance and control are violated, and attempts are made to explain this violation by both victims and others. The theme of sexuality myths includes negative and incorrect beliefs that a man who is sexually assaulted by another male must therefore be gay (Stermac et al., 2004), or have been acting in a ‘gay manner’ (Coxell and King, 2010), or that *only* gay men are raped (Hillman, O'Mara, Taylor-Robinson and Harris, 1990; Laurent, 1993; Struckman-Johnson, 1991). Confusingly and in complete contradiction, other myths state that gay men *cannot* be sexually assaulted, as the act of anal penetration itself is ‘homosexual by nature’, and thus cannot be non-consensual (Cotton, 1992), and that gay men constitute ‘willing’ victims (Mezey and King, 1989). These erroneous attitudes are propagated and reinforced by still extant homophobia within society, which promotes a range of negative responses to both consensual and non-consensual sex between men, from disapproval and disbelief, to disgust and violence (Ahmad and Bhugra, 2010). Indeed, such is the societal prejudice towards sexual minority men, that some myths suggest that gay or bisexual men ‘deserve’ their victimisation, as a reward for a ‘deviant’ lifestyle (Turchik and Edwards, 2012) and that they have ‘brought it upon themselves’ (Cotton, 1992). Ironically, evidence that suggests that the vast majority of male rape victims (approximately 80%) identify as heterosexual (Groth and Burgess, 1980; Isley & Gehrenbeck-Shim, 1997). Though, it must be noted that such individuals are often believed to be hiding ‘secret sexual desires’ and have thus claimed rape to hide their illicit activities (King, 1992); a myth likely underpinned by broader misconceptions of rape as motivated by sexual desire, rather than dominance and power (Hickson et al., 1994). Taken together, these myths are mostly representative of broader negative beliefs around sexual minority men; themselves outdated, bigoted, and fundamentally incorrect.

Other myths related to sexual desire are those related to pleasure and men's physiological responses to sexual activity (consensual or otherwise). Specifically, widely held beliefs that physiological reactions to physical stimulation and arousal (e.g., erection and/or ejaculation) imply pleasure and enjoyment (McMullen, 1990), are problematic for victim-survivors, as this may lead them to question whether the event was rape, or to feel as if their body has ‘betrayed them’ (Coxell and King, 2010; Gonsiorek, 1994; Sarrell and Masters, 1982). Such myths also encourage others to assume enjoyment, and thus disbelief in claims of rape, in a similar manner to female victims; where vaginal lubrication, and even orgasm as a protective bodily response, is misinterpreted as pleasure (Suschinsky and Lalumière, 2010). This is despite research which states that men frequently achieve an erection and/or ejaculation during an assault and that such reactions may be a generalised bodily reaction to extreme emotional turmoil (Sarrell and Masters, 1982). Arousal myths and sexuality myths also heavily related, as the supposed ‘enjoyment’ of anal or oral penetration, as implied by an erection or ejaculation, trigger associated homophobic beliefs.

A specific subset of myths relate to the behaviour and nature of perpetrators, many of which mirror sexuality myths relating to victims. Specifically, as with male victims, assumptions are made that men who rape other men are gay (Groth and Burgess, 1980; McMullen, 1990; Mezey and King, 1987; Struckman-Johnson, 1991) and are acting upon either secret or overt sexual desires (Coxell and King, 2010). Again, this is despite evidence suggesting that approximately 90% of perpetrators identify as heterosexual (Isley & Gehrenbeck-Shim, 1997). These perpetrator specific myths are again most likely explained by misunderstandings concerning motivations for rape (e.g., that male rape is motivated by sexual desire, rather than agreed upon dominance explanations) and male sexuality (e.g., that only exclusively gay men are interested in sexual interactions with other men).

Context myths broadly relate to disbelief concerning the existence of male rape at all, a phenomenon exacerbated by a lack of visibility for male victims within political, social and academic spheres. Indeed, the myth that male rape is exceptionally rape, if it occurs at all (Mezey and King, 1989; Scarce, 1997; Struckman-Johnson and Struckman-Johnson, 1992), is informed by all other myths outlined above which seek to maximise disbelief in its occurrence and likelihood. Such myths are also contrary to the information and data available on the prevalence of male rape, both within the UK (~12,000 per annum, Office for National Statistics, 2018), and countries worldwide (e.g., the US, ~131,000 per annum, Stemple and Meyer, 2014). Other context myths seek to suggest that male rape, when it does occur, only happens in *particular* contexts, and that this occurrence is typical or inevitable. These include prisons, the military, LGBTQ + venues, and male societies (e.g., sports clubs or fraternities; Garnets et al., 1990; Kaufman, 1984; Lacey and Roberts, 1991; Scarce, 1997; Turchik and Edwards, 2012). It is principally within this genre of myth that ‘traditional’ rape myths are applied to men. For example, men, similarly to women, are judged to be more responsible for their victimisation if they have consumed alcohol or drugs, thus compromising their ability to control and consciously participate in sexual interactions (Sleath and Bull, 2010). It is worth noting however that applications of myths are never identical, as, whilst some elements are similar (i.e., consumption of alcohol having a detrimental effect on memory in both men and women), other elements are shaped by sex-specific expectations and norms (i.e., men's consumption of alcohol as compromising their strength and ability to fight off an attacker).

Finally, several myths relate to the effect (or lack thereof) of rape on men. Specifically, myths articulate that men are not psychologically or physically affected by rape, principally because they are men and can ‘take it’, and that they are not as affected by the incident as women (Struckman-Johnson and Struckman-Johnson, 1992). This is despite growing literature exploring and highlighting the profound psychological and physical effects of rape on men, and their need for belief and care (Goyer and Eddleman, 1984). Indeed, research has demonstrated that men who have had sexually coercive experiences as an adult are more likely (than those who have not) to experience a range of psychological problems, such as lower self-esteem, increased depressive symptoms, suicidal ideation and self-harm behaviours, anxiety and post-traumatic stress symptoms, substance abuse and dependence issues, sexual dysfunction and identity confusion (Turchik and Edwards, 2012). Alongside masculinity myths described above, it is within effect myths that notions of shame are most explored (i.e., that men should feel shame following their victimisation, for ‘allowing’ themselves to ‘become’ victims; Struckman-Johnson and Struckman-Johnson, 1992). This attribution of shame is similar to that experienced by female rape victims, with additional attributions resulting from male gender role norm violations (i.e., showing vulnerability). Subsequently, men are expected to be able to cope on their own following victimisation (Struckman-Johnson, 1991). Thus, taken together, whilst more research on male rape myths is needed, previous studies have at least provided some information on the nature of such myths.

### 1.3 The influence of male rape myths

As knowledge of male rape myths has increased, a limited number of studies have emerged exploring their effect on judgments towards male victims and perpetrators, most commonly through attribution of responsibility ratings. For example, several studies demonstrate that gay victims are attributed higher levels of responsibility for their victimisation than heterosexual victims (see Davies and Rogers, 2006, for review). Subsequently, it has been reasoned that participants are informed by homophobic attitudes and sexuality male rape myths, in making their judgments (M. Davies and Rogers, 2006). Moreover, several studies have demonstrated how myths interact, for example by finding that participants worryingly believe that gay male victims find their assault more pleasurable, and that they suffer from less trauma (Michelle Davies, Pollard and Archer, 2001; Mitchell et al., 1999). The influence of masculinity myths are shown through studies exploring resistance, where higher responsibility attributions are awarded to men who ‘fail’ to resist their attacker (Howard, 1984a, 1984b). More broadly, several studies show that men are attributed greater responsibility for their victimisation than women (I. Anderson and Quinn, 2009), particularly when judged by other men (White and Kurpius, 2002). Such results suggest that participants are more blaming of men due to the effects myths which present male rape as unlikely, and a consequence of victim behaviour. Male rape myths have also been shown to be related to a number of demographic variables (i.e., males have higher adherence; see Walfield, 2018 for review), and several proximal attitudes, including measures of traditional rape myths, traditional gender roles, negative attitudes towards sexual minority men, stereotypes about masculinity and male inexpressiveness, and the endorsement of traditional male gender roles (Chapleau et al., 2008; M. Davies, Gilston and Rogers, 2012; Kassing et al., 2005; Melanson, 1999; Nalavany and Abell, 2004; Walfield, 2018).

However, whilst some information on the influence of male rape myths is available, the research outlined above remains limited, as the effects of only some male rape myths are explored. Moreover, information regarding the prevalence of male rape myth acceptance within society, and our understanding of the relationship between male rape myth acceptance and judgments given, is currently extremely inadequate. Moreover, levels of MRMA in specialist populations who directly interact with male victims (such as counsellors, service providers, police officers etc.) is also yet to be comprehensively measured. The principal reason for the scarcity of literature in this area is the lack of a reliable and targeted scale measuring said myths.

### 1.4 Current measures of male rape myths

At present, two scales for the measure of male rape myth acceptance exist. The first is a 12-item scale developed by Struckman-Johnson and Struckman-Johnson, 1992. This scale is constructed of six items each with a male and female perpetrator version, with two items measuring each of these three general beliefs: (a) male rape does not happen (e.g., “it is impossible to rape a man”), (b) rape is the victim's fault (e.g., “men are to blame for not escaping”), and (c) men would not be traumatised by rape (e.g., “men do not need counselling after being raped”). Respondents self-rate their beliefs on a 6-point Likert scale ranging from 1 (strongly disagree) to 6 (strongly agree).

Several issues with this scale present. First, questions relating to incidents involving female perpetrators are included. This is problematic as it can be argued that whilst there are some areas of overlap (e.g., victimisation as a threat to masculinity, misinterpretation of physical arousal as pleasure), many myths about men raped by other men and about men sexually assaulted by women, are distinct and should be measured as such. Take, for example, myths around sexuality which only exist in relation to male-on-male assaults, or myths around perpetrators which by nature must be sex-specific. A further example is the common myth concerning female-perpetrated sexual assault, that ‘men always want sex’ (Clements-Schreiber and Rempel, 1995), which would carry very different conceptualisations in the context of a man raped by another man. It is thus argued that there is a strong theoretical basis for the separate investigation and measurement of male-on-male and female-on-male myths, as they evoke very different affective and cognitive interpretations and reactions. Indeed, Walfield (2018) argues that a gender neutral approach to these kind of measurements is to be avoided, as it invites the risk of erasure of gender-specific experiences. Indeed, there are several dedicated organisations across the UK which specifically focus on providing support to men assaulted by other men, to whom research utilising such a scale would be beneficial (though it should be noted that an additional scale/sub scale assessing female-on-male rape/sexual assault would be beneficial in the future). Additionally, it is important to note that traditional rape myth measures (e.g., IRMAS) specifically assess attitudes towards acts of male-on-female rape only (i.e., they do not focus on female perpetrated sexual assault). Thus, as a starting point, a measure constructed to measure myths surrounding rape of males would, in the UK, have a justifiably similar focus of only male-on-male incidents.

The second issue is that the range of myths measured is too narrow and does not capture the variety of beliefs regarding male rape. As outlined above, there are (at least) six principal male rape myth ‘themes’; this scale only covers three (with only two items each). The final issue is that measures of scale reliability are unavailable. Cronbach's alpha values are not reported in the original publication (Struckman-Johnson and Struckman-Johnson, 1992) or in most subsequent studies utilising the scale (e.g., Chapleau et al., 2008).

The second available scale is a 22-item questionnaire, developed as part of a doctoral dissertation programme by Melanson (1999), designed to measure false, stereotypical, or prejudicial beliefs about male rape. Sample items include “male rape is usually committed by homosexuals” and “a man who has been raped has lost his manhood”. As above, participants answer using a 6-point Likert scale ranging from 1 (strongly disagree) to 6 (strongly agree).

Melanson's scale improved upon the Struckman-Johnson and Struckman-Johnson measure in two ways. First, several reliability analyses are available, with this scale demonstrating excellent reliability scores (.90 in the original research, and between .85 and .91 when utilised in subsequent studies, e.g., Kassing et al., 2005). Second, a greater variety of questions assess all but one of the themes outlined above. However, despite these advancements, five significant issues remain. First, as stated, not all the myth themes described above are measured in detail (i.e., there are no questions that ask about the actions of perpetrators, and only two questions measure beliefs about the effect of rape on male victims). Second, this scale still includes questions regarding female perpetrators, which, as argued above, is problematic. Third, the sample used to validate this measure is both too small (only 303 participants were utilised to assess and validate *80* potential items) and too homogenous (only undergraduate students were utilised). Fourth, this scale was still developed over 20 years ago, when attitudes towards male victims were likely different to the present day, and, as such, would potentially have been phrased differently. Finally, the publication detailing this scale has not been peer-reviewed (though it should be noted that subsequent studies utilising the scale have been), and it can thus be argued that the process for development and determination of reliability of this scale has not been subjected to rigorous academic appraisal. In summation, whilst some measures of male rape myth acceptance do exist, they require improvement in a number of key areas.

### 1.5 The present study

As detailed above, since the early 1990s, interest and research in the existence of male rape myths has significantly increased. Such research has detailed the nature of said myths and has begun to examine the influence of such attitudes on reactions and judgments toward male victims. However, at present, research is limited by the lack of a reliable, comprehensive, and targeted tool to measure acceptance of male rape myths, as exists for ‘traditional’ rape myths. The necessity for such a scale is outlined well in Struckman-Johnson and Struckman-Johnson, 1992 paper:

“The ultimate goal of research on this topic… is the investigation of the relationship between male rape myth acceptance and underreporting of male sexual assault… If rape myth acceptance can be documented, one can then determine if beliefs are indeed related to reporting, to treatment, and to justice received by male victims. At a minimum, research will stimulate awareness of the problem and encourage development of programs to counteract cultural misunderstandings of male rape” (p. 98)

Arguably, this need is most exemplified within criminal justice contexts, as increasing our understanding of how specialist populations (e.g., police officers) interact with male victims is critical in improving victim engagement and satisfaction with justice processes, and case success.

The aim of the present research therefore was to produce and validate the first Male Rape Myth Acceptance Scale (MRMAS), measuring myths under six themes: masculinity, sexuality, pleasure, perpetrators, context, and effect. Study 1 details the formulation, analysis, and selection of male rape myth items through a variety of reliability measures including exploratory factor analysis. Study 2 describes validation of a revised 44-item scale, including assessment of validity in relation proximal constructs.

## 2 Study 1

### 2.1 Method

Full ethical approval for this study was granted by the University Research and Ethics Committee at the University of West London.

#### 2.1.1 Scale development

As described by Clifton (2019), when approaching scale development, an inevitable trade-off between validity and reliability must occur. Such battles are fought in a number of areas, ranging from the wording and content of items, to their administration and scoring. As such, Clifton recommends that authors are forthright in their priorities, so that reviewers may hold their processes to account (2019). As such, we make it clear here that our goal was to produce a highly reliable measure, that preserved content validity wherever possible. As such, as outlined below, decisions regarding item *generation* were designed to ensure maximum validity, with decisions taken during item *administration and analysis* then designed to maximise reliability, which was subsequently prioritised. These choices were made as this study was not designed to explore which myths exist (i.e., theoretically exploratory), but rather to develop a robust system of measurement (i.e., methodologically exploratory). Thus predictive, rather than content validity was prioritised (Clifton, 2019).

To formulate scale items, the authors engaged in over 60 h of literature review and formulated discussions. Some deliberations were between just the authors, whilst others were alongside research assistants and departmental colleagues, undergraduate students, or with family and friends (acting as representative of the general public). Discussions were either theory-led, drawing from existing literature on male rape myths (i.e., to measure one of the six myth ‘themes’), or more informal, for example by asking others what they thought about male rape (e.g., “When you think of male rape, what comes to mind?”). During item generation, careful consideration of the balance between reliability and validity was considered, as outlined in Clifton (2019). Specifically, to maximise validity: item content was kept as diverse as possible, whilst still being theoretically informed; item difficulty was moderate to ensure even levels of accessibility; and items were constructed using varied terminology, again, to ensure even levels of accessibility (Clifton, 2019). Items were initially specifically generated to measure one of the six initial male rape myth categories, and were labelled appropriately: Masculinity (M), Sexuality (S), Pleasure (PL), Effect (E), Context (C), and Perpetrators (P). They were also classified as to whether they represented a behavioural (B), characterological (C) or Typological (T) judgment, and this constituted the second letter in the item label. An additional letter was added to item labels based on whether they represented a cognitive (C) or affective (A) evaluation. Each of the six principal categories had between six and eleven questions (with numbers of items determined by authors judgement of appropriate theoretical coverage). Some items were also reversed to guard against acquiescence bias, though this was not excessive so as to preserve validity, but not compromise reliability (as outlined by Clifton, 2019).

The process outlined above resulted in the first iteration of the Male Rape Myth Acceptance Scale (MRMAS). This constituted 55 items covering six areas of male rape myths, as outlined above, including: Masculinity (e.g., “I find it difficult to believe one man could sexually overpower another man”), Sexuality (e.g., “Male on male rape only happens to homosexual men”), Pleasure (e.g., “A male victim who ejaculates during the incident has not been raped”), Effect (e.g., “Male victims of rape are not traumatised by the incident”), Context (e.g., “Almost all male rape occurs in institutions such as prisons or the military”), and Perpetrators (e.g., “A man would not rape another man if he was sexually fulfilled elsewhere”). Participants answered on a 7-point Likert scale between 1 (Strong Disagree) and 7 (Strongly Agree), as used in other popular rape myth scales (i.e., the AMMSA). Items were presented in the same order to all participants, and in unidimensional blocks (as reliability was prioritised during administration; Clifton, 2019).

#### 2.1.2 Participants

Participants were 510 undergraduate students (M = 24.08, SD = 6.63, min = 18.00, max = 58.00, 295 female) from two universities in the UK; one in London, and one in the North West (though this was not recorded during participation). Participants’ identified gender was predominantly as a woman (53.1%) or a man (33.7%), with male (6.9%) and female (4.3%) constituting the next highest percentages. Other responses included agender, cisgender, fourth gender and genderqueer (all between .2 and .6%). Most participants identified as heterosexual (81.4%), with 8.4% identifying as bisexual, 5.1% as homosexual, and 4.5% preferring not to say. The sample also included a variety of ethnic backgrounds, with White participants constituting 47.1%, Black/Black British providing 21.8%, and Asian/Asian British making up 16.3%. Other and Mixed groups constituted the remaining 14.9%. Participants were recruited through opportunity sampling across the two institution campuses. All participants were offered the chance to be entered into a prize draw to win a £25 Amazon voucher, and psychology students were also awarded two research credits, as part of departmental initiatives to encourage research participation.

#### 2.1.3 Procedure

Participants were recruited using two principal methods. Some were recruited through advertisement in lectures at their institution and were provided a link to the study should they wish to take part. Others were approached by a research assistant whilst they were alone and in a quiet space (such as a library) and were asked to either complete the survey on a tablet or take a leaflet to do so in their own time. Participants were made aware of the general context of the study (i.e., that they would be answering questions pertaining to male-on-male rape), before being asked if they would like to participate. If they clicked on the link provided, they were presented with an information sheet outlining the study in more detail, before being asked to provide informed consent – which was obtained from all participants. They were then asked to provide basic demographic information, before being presented with brief instructions on how to complete the MRMAS, followed by the questionnaire. Once complete, participants were presented with a debrief explaining the purpose and aims of the study and directing them to appropriate support services should they be needed. Participants could also give their contact information (stored separately to their data) to be entered into the prize draw. This study was approved by the University Research and Ethics Committee (UREC) at the University of West London.

### 2.2 Results

Using SPSS Version 25 descriptive statistics and item-to-total correlations were produced to allow for assessment of sample distribution, and to provide guidance on which items required elimination for Study 2. Exploratory factor analysis was performed using SPSS to assess the latent structure of the scale and further identify items which did not load well, and thus could also be eliminated. The *Psych* package in R (Revelle, 2017) was used to perform the Scree test, Parallel analysis, the Very Simple Structure (VSS) and MAP tests (Horn, 1965; Revelle and Rocklin, 1979; and Velicer, 1976; all cited in Revelle, 2017) to help determine the number of factors to extract. The *Psych* package was also used to calculate reliability estimates (Cronbach's Alpha, Hierarchal Omega and Omega Total).

#### 2.2.1 Descriptive statistics and reliability estimates

The MRMAS full scale mean (average across all 55 items) for individual participants ranged from 1.22 to 5.91 (Range = 4.69), with a sample MRMAS full scale mean of 2.59. A skewness value of 0.77 suggests moderate positive skew in the MRMAS full scale mean. This is unsurprising, as scales of this nature, which ask questions on sensitive or emotive topics such as rape, often produce lower means. A kurtosis value of 0.26 in the MRMAS full scale mean suggests a normal level for a sample of this size (Field, 2009). The Cronbach's Alpha for all 55 items was .97, while the Hierarchal Omega/Omega total coefficients were .88/.97 respectively when calculated using Maximum Likelihood estimation with Pearson correlations. A Promax rotation was applied in this analysis to account for the possibility of more than one factor existing in the data. An oblique rotation was chosen because any latent factors should still be representative of rape myths, and therefore should be significantly related. As the response options for each item in the measure can be viewed as continuous ordered categories (Schmidt, 2011), this analysis was also repeated using Polychoric correlations instead of Pearson correlations to estimate the reliability coefficients (Schmidt, 2011). This suggested the Cronbach's Alpha for all 55 items was .98, while the Hierarchal Omega/Omega total coefficients were .75/.98 respectively. These demonstrate overall reliability for a single general factor solution, although the discrepancy in the Hierarchal Omega and Omega total coefficients suggests some of the variance in the 55 items could be attributable to a multiple factor structure or specific item variances.

Individual item descriptive statistics and item-total correlations are shown in Table 1. Overall, item means sat towards the middle to lower part of the Likert scale, and a number of moderate to high positive skewness values were observed suggesting the data were somewhat skewed. However, most item-total correlations demonstrated that, despite several ‘lower’ mean scores, most items correlated strongly to the total. Thus, considering the commonality of positive skew for scales of this type, when identifying extreme outlier candidates for elimination rather than set the arbitrary threshold for elimination at mean < 2, the decision was taken to identify just the three items with the lowest mean score (shown in bold). The two items which showed weak item-total correlations (shown in italics) were also identified for elimination. One final candidate item was identified after feedback from research assistants suggesting a lack of understanding of terminology from participants (specifically, the word ejaculation; item underlined), with no alternative phrasing possible. All such decisions were taken to maximise reliability at the analysis stage (Clifton, 2019).

**Table 1:** Descriptive statistics for each MRMAS item – study 1.

Item	Code	Mean	SD	Item-Total correlation	Skewness
1. If a man is raped it does not mean he is weak	1M.C.A.R	2.69	2.18	.283	1.036
2. A man who is raped must have been behaving in a way that made him appear homosexual	1S.B.A	1.84	1.35	.568	1.856
3. I would be less inclined to believe a man who said he had been raped if he got an erection during the incident	1PL.B.A	2.39	1.59	.616	1.026
4. Heterosexual men are more traumatized by their experience of being raped than women	1E.C.A.	3.17	1.81	.421	0.365
5. Male rape is very rare, if it occurs at all	1C.T.C.	3.15	1.73	.534	0.449
6. Heterosexual men who commit rape against other men do so to assert their dominance	1P.B.C.R.	3.91	1.69	.251	0.292
7. I find it difficult to believe one man could sexually overpower another man	2M.C.A.	2.26	1.59	.649	1.362
8. Male on male rape only happens to homosexual men	2S.C.C.	1.92	1.30	.651	1.849
9. A male victim who ejaculates during the incident has not been raped	2PL.B.C.	2.13	1.43	.672	1.335
**10. Male victims of rape are not traumatized by the incident**	2E.C.A.	**1.55**	1.03	.624	2.529
11. Almost all male rape occurs in institutions such as prisons or the military	2C.T.C.	2.85	1.58	.539	0.622
12. A homosexual man who rapes other men does so out of sexual desire	2P.B.C.	3.60	1.67	.443	0.052
13. Most men would be able to fight off a male sexual attacker	3M.C.C.	2.96	1.66	.678	0.613
14. Rape is an accepted risk of a ‘homosexual lifestyle’	3S.B.A.	1.96	1.38	.682	1.509
15. During a sexual attack it is reasonable for the victim's erection to be viewed as consent	3PL.B.A.	2.09	1.44	.687	1.225
16. Without physical trauma, I would be less included to believe a man had been raped	3E.C.A.	2.09	1.39	.636	1.407
17. The idea of a man being raped is somewhat amusing	3C.C.A	1.76	1.45	.562	2.010
18. Heterosexual men who commit rape do so to act upon secret homosexual desires	3P.C.C.	3.44	1.69	.497	0.121
19. In ‘real’ cases of male rape, there will be some evidence of physical resistance	4M.B.C.	3.60	1.81	.506	0.064
20. Heterosexual men ‘cry rape’ to hide their homosexual activities	4S.B.A.	2.72	1.54	.649	0.456
21. Even if force is used to initiate sex, the victim's erection can be interpreted as pleasure	4PL.B.C.	2.47	1.56	.635	0.889
22. I would expect heterosexual victims of rape to be more traumatized than homosexual victims	4E.C.A.	2.74	1.89	.659	0.758
23. Coercive sexual practices between men (e.g., forced oral sex) form a legitimate part of group initiations such as those used in fraternities or sporting societies	4C.T.C.	2.73	1.55	.555	0.343
24. A man would not rape another man if he was sexually fulfilled elsewhere	4P.B.C.	2.57	1.71	.670	0.908
**25. A man who fails to escape a sexual attack is partially responsible for his rape**	5M.B.A.	**1.64**	1.21	.670	2.245
26. Just because a man is raped does not mean he is homosexual	5S.C.C.R	2.08	1.70	.225	1.794
27. If a man is being sexually attacked, his ejaculation is proof he found the experience somewhat pleasurable	5PL.B.A.	2.22	1.46	.699	1.040
28. Men should feel ashamed as a result of being raped	5E.C.A.	1.97	1.62	.596	1.789
29. Most cases of male rape include the use of a weapon	5C.T.C.	3.54	1.35	.330	-0.182
30. Male rape is only perpetrated by homosexual men	5P.C.C.	2.18	1.41	.741	1.286
*31. For a man, not resisting a sexual attack from another man, is a reasonable response*	6M.B.A.	4.94	1.65	-.154	-0.378
**32. A man who is raped must be homosexual even if he claims to be heterosexual**	6S.C.A.	**1.74**	1.25	.679	1.900
33. A homosexual man who has been raped probably enjoyed the experience to some extent	6PL.C.A.	1.88	1.31	.748	1.617
34. Homosexual men are more traumatized by their experience of being raped than women	6E.C.A.	2.55	1.52	.476	0.618
35. A man is more responsible for his own rape if he frequents a known homosexual area or establishment	6C.B.A.	2.05	1.42	.791	1.279
36. Only men who are big and strong are able to rape other men	6P.C.C.	2.13	1.39	.692	1.274
37. I would find it difficult to consider a man a ‘real man’ if he said he had been raped	7M.C.A.	1.89	1.42	.731	1.733
38. If a man has already had consensual sex with other men, I would not believe his claims of rape	7S.B.A.	2.06	1.48	.765	1.468
39. A man who is raped is not as traumatized by the experience as a woman	7E.C.A.	2.22	1.52	.646	1.132
40. If a man is drunk or taking drugs he is accepting rape as a possible risk	7C.B.A	2.26	1.63	.684	1.108
41. Men who commit rape are naturally more aggressive in their day to day lives	7P.C.C.	3.40	1.64	.424	0.057
42. It is acceptable for a ‘real man’ to show fear during a sexual attack by another man	8M.B.A.	2.57	1.81	.301	1.149
43. A man who claims to have been raped probably just changed his mind after initially consenting to sex	8S.B.A.	2.29	1.42	.714	0.983
44. A male victim's reaction to rape is more likely to be practical than emotional (e.g., obtaining a HIV test rather than seeking support)	8E.B.C.	3.54	1.75	.356	-0.002
45. A male victim of rape must have behaved in a way that invited the assault	8C.B.A.	2.02	1.38	.762	1.335
*46. Raping another man is not a sign of mental illness*	8P.C.A.R.	4.66	1.87	-.040	-0.365
47. A heterosexual man who had been raped would still be desirable to women	9M.C.A.R.	2.75	1.62	.303	0.934
48. Male rape is a homosexual act	9S.C.C.	2.91	1.85	.574	0.639
49. If a man has been raped he should be able to cope on his own	9E.C.A.	1.99	1.33	.667	1.383
50. I would find it difficult to believe a man had been raped if he had previously consented to sex with the same man	9C.B.A.R.	2.40	1.59	.741	1.038
51. Regardless of how they identify themselves, I believe that men who rape other men are homosexual	9P.C.A	2.91	1.81	.590	0.636
52. ‘Real men’ cannot be raped	10M.C.A.	1.76	1.34	.757	2.123
53. I would expect a man to be ‘matter of fact’ and in control of his emotions when reporting a rape	10E.B.A.	2.36	1.53	.694	1.012
54. A man who has been raped did not set sexual limits understood by the perpetrator	10C.B.C.	2.45	1.51	.657	0.761
55. Male victims of rape have very little emotional trauma to cope with	11E.C.A.	1.85	1.36	.698	1.735

Note: Skewness SE = 0.108. _a_Code is comprised of a) a number denoting the position of that item within each theme, b) a letter denoting the theme (M = Masculinity, S = Sexuality, PL = Pleasure, E = Effect, C = Context, P = Perpetrator), c) whether the item is Behavioral (B), Characterological (C) or Typology (T), and d) whether the item is Affective (A) or Cognitive (C). If the item also has an R at the end of its code, this means the question is reverse scored. _b_Items shown in bold were identified for elimination due to low item means. _c_Items shown in italics were identified for elimination due to weak item-total correlations. _d_Underlined items were eliminated due to feedback suggesting participant lack of understanding of terminology.

#### 2.2.2 Exploratory factor analysis

All items were subjected to a Principal Axis Factoring exploratory factor analysis (EFA), to assess the underlying structure of the scale, and to identify further candidate items for elimination (under recommendations from Costello and Osborne, 2005; Fabrigar et al., 1999; Russell, 2002). Principal Axis factoring was used due to the positive skew displayed by a number of items measured in the scale (see Table 1). The use of this extraction technique has been advised in cases where a number of the items display a non-normal distribution, as the estimation technique does not require normal distribution assumptions to be met (Costello and Osborne, 2005; Fabrigar et al., 1999).

Inspection of the correlation matrix revealed the presence of many coefficients of .30 and above. The Kaiser-Meyer-Olkin value of .97 exceeded the recommended value of .5, and is described as ‘great’ (Kaiser, 1970, 1974) suggesting that the sample size was sufficient. Furthermore Bartlett's Test of Sphericity (Bartlett, 1954) reached statistical significance, supporting the factorability of the correlation matrices. Using Kaiser's criteria of eigenvalues of >1 alone to retain factors was not appropriate here, as variable communalities (after extraction) did not consistently reach above .60 (Field, 2009). Using the scree plot, parallel analysis, the MAP and VSS tests, and inspecting cumulative variance explained, this suggested either a two factor (scree plot, parallel analysis) or three factor (MAP, VSS) solution. Both solutions were run, with the two factor model explaining 41.66% of the variance in the 55 items measured, while the three factor model explained 43.70%. A Promax rotation was then applied to the factors. Promax rotation specifically was used upon recommendations from the EFA literature (Fabrigar et al., 1999; Russell, 2002), including the technique being more efficient in determining an optimal rotated solution through fewer iterations in large scale datasets. As the MAP and VSS tests indicated a very limited impact of the addition of the third factor, which only explained 2% of the variance with only three items loading out of 55 (26, 42 and 47, which all tapped into maintaining the heteronormativity of the victim), it was decided the two factor solution would be the most appropriate to ensure the stability of the Study 1 data solution. Factor loadings for each item are presented in Table 2. As recommended by Field (2009), a suppression threshold of .4 was chosen as this was an exploratory study.

**Table 2:** Principal Axis Factoring factor loadings for each MRMAS item, following Promax rotation – study 1.

Item	Factor 1 – Blame	Factor 2 – Minimisation/Exoneration
26. Just because a man is raped does not mean he is homosexual	.830	-.614
42. It is acceptable for a ‘real man’ to show fear during a sexual attack by another man	.816	-.518
55. Male victims of rape have very little emotional trauma to cope with	.755	
32. A man who is raped must be homosexual even if he claims to be heterosexual	.750	
10. Male victims of rape are not traumatized by the incident	.733	
25. A man who fails to escape a sexual attack is partially responsible for his rape	.729	
52. ‘Real men’ cannot be raped	.724	
45. A male victim of rape must have behaved in a way that invited the assault	.712	
47. A heterosexual man who had been raped would still be desirable to women	.700	-.404
38. If a man has already had consensual sex with other men, I would not believe his claims of rape	.683	
33. A homosexual man who has been raped probably enjoyed the experience to some extent	.639	
37. I would find it difficult to consider a man a ‘real man’ if he said he had been raped	.633	
49. If a man has been raped he should be able to cope on his own	.621	
35. A man is more responsible for his own rape if he frequents a known homosexual area or establishment	.586	
14. Rape is an accepted risk of a ‘homosexual lifestyle’	.564	
8. Male on male rape only happens to homosexual men	.554	
43. A man who claims to have been raped probably just changed his mind after initially consenting to sex	.542	
28. Men should feel ashamed as a result of being raped	.530	
1. If a man is raped it does not mean he is weak	.511	
39. A man who is raped is not as traumatized by the experience as a woman	.500	
36. Only men who are big and strong are able to rape other men	.480	
2. A man who is raped must have been behaving in a way that made him appear homosexual	.466	
53. I would expect a man to be ‘matter of fact’ and in control of his emotions when reporting a rape	.446	
30. Male rape is only perpetrated by homosexual men	.434	
9. A male victim who ejaculates during the incident has not been raped	.423	
40. If a man is drunk or taking drugs he is accepting rape as a possible risk	.421	
18. Heterosexual men who commit rape do so to act upon secret homosexual desires		.850
19. In ‘real’ cases of male rape, there will be some evidence of physical resistance		.773
12. A homosexual man who rapes other men does so out of sexual desire		.696
29. Most cases of male rape include the use of a weapon		.657
20. Heterosexual men ‘cry rape’ to hide their homosexual activities		.654
22. I would expect heterosexual victims of rape to be more traumatized than homosexual victims		.643
13. Most men would be able to fight off a male sexual attacker		.633
5. Male rape is very rare, if it occurs at all		.617
48. Male rape is a homosexual act		.611
51. Regardless of how they identify themselves, I believe that men who rape other men are homosexual		.594
41. Men who commit rape are naturally more aggressive in their day to day lives		.594
11. Almost all male rape occurs in institutions such as prisons or the military		.583
44. A male victim's reaction to rape is more likely to be practical than emotional (e.g., obtaining a HIV test rather than seeking support)		.550
21. Even if force is used to initiate sex, the victim's erection can be interpreted as pleasure		.512
24. A man would not rape another man if he was sexually fulfilled elsewhere		.506
23. Coercive sexual practices between men (e.g., forced oral sex) form a legitimate part of group initiations such as those used in fraternities or sporting societies		.496
54. A man who has been raped did not set sexual limits understood by the perpetrator		.495
50. I would find it difficult to believe a man had been raped if he had previously consented to sex with the same man		.439
27. If a man is being sexually attacked, his ejaculation is proof he found the experience somewhat pleasurable		.435
3. I would be less inclined to believe a man who said he had been raped if he got an erection during the incident		.415
4. Heterosexual men are more traumatized by their experience of being raped than women		.412

16. Without physical trauma, I would be less included to believe a man had been raped		.412
**6. Heterosexual men who commit rape against other men do so to assert their dominance**		
**7. I find it difficult to believe one man could sexually overpower another man**		
**15. During a sexual attack it is reasonable for the victim's erection to be viewed as consent**		
**17. The idea of a man being raped is somewhat amusing**		
**31. For a man, not resisting a sexual attack from another man, is a reasonable response**		
**34. Homosexual men are more traumatized by their experience of being raped than women**		
**46. Raping another man is not a sign of mental illness**		

Note Items shown in bold were identified for elimination due to a lack of substantial loading on either factor.

A two-factor solution demonstrated near simple structure with most items loading onto one of the two factors. Upon closer inspection, it appears that, whilst some overlap occurs, most of the items loading onto factor one are principally representative of attitudes which a) directly blame the victim, and/or b) a suggest that only certain groups of men (in this case, gay or weak men) are raped. Items which load onto factor two appear to be primarily representative of attitudes which a) exonerate the perpetrator, and/or b) minimise the incident itself. The grouping of items in this way suggests that, whilst they are all representative of male rape myths, there may be a latent delineation between two distinct subsets of myth; *Blame* versus *Minimisation/Exoneration*. The two factors were found to be strongly positively correlated in the two factor solution (*r* = .76).

Seven items did not load above .40 onto either factor (6, 7, 15, 17, 31, 34, 46), suggesting that they were a) answered significantly differently to other items, b) measuring an unrelated concept, or both. These items were therefore identified for elimination for Study 2 and the final scale. In addition, any items loading below .42 onto a factor (items 3, 4 and 16) were identified for elimination to ensure only securely (not borderline) loading items were taken forward for the study 2 scales(s). Finally the remaining four items identified for elimination through the descriptive statistics and item-total correlations as above (items 10, 25, 27, 32) were removed. Again, all such decisions were taken as reliability was prioritised during analysis (Clifton, 2019). This left 41 items to be taken over into study 2 to assess the validity and reliability of the measure in a second sample. The reliability estimates for the 41 item model using Maximum Likelihood estimation with Pearson (Polychoric) correlations and a Promax rotation were Alpha = .96 (.97), Hierarchal omega = .89 (.71), Omega total = .97 (.98).

## 3 Study 2

A second study was then conducted to provide reliability and validity measurement for the new, refined MRMAS scale, utilising a more representative population. This included measurement of the relationship between the new MRMAS scale, and related, proximal constructs previously shown to have an association with male rape myth acceptance, including both demographic (i.e., age) and attitudinal variables (i.e., homophobia; see Walfield, 2018 for review).

### 3.1 Method

#### 3.1.1 Participants

528 individuals (who did not take part in Study 1) took part in study 2 as part of two groups. First, undergraduate students (N = 346, M = 22.97yrs, SD = 5.00, min = 18.00, max = 49.00, 214 female) from two universities in the UK; one in London, and one in the North West (though this was not recorded during participation). Second, members of the general population (N = 182, M = 35.39yrs, SD = 10.95, min = 19.00, max = 68.00, 110 female). Information on participants sex, identified gender, sexuality, ethnicity, employment status, and profession are detailed in Table 3. All participants were offered the chance to be entered into a prize draw to win a £25 Amazon voucher, and psychology students were also awarded two research credits, as part of departmental initiatives to encourage research participation.

**Table 3:** Demographic information for participant groups in study 2.

		Undergraduate Students		General Population	
Age	Mean	22.97		35.39	
	SD	5.00		10.95	
	Min	18.00		19.00	
	Max	49.00		68.00	
Income	Median	N/A		£30,000-£40,000	

		*N*	*%*	*N*	*%*
Sex (%)	Male	132	38.2	74	40.2
	Female	214	61.8	110	59.8
Identified Gender (%)	Woman	196	56.6	100	54.3
	Man	114	32.9	61	33.2
	Female	17	4.9	6	3.3
	Male	9	2.6	11	6.0
	Other	10	3	6	3.2
Sexuality	Heterosexual	271	78.3	134	72.8
	Homosexual	11	3.2	15	8.2
	Bisexual	32	9.2	28	15.2
	Asexual	9	2.6	1	0.5
	Prefer Not to Say	23	6.6	6	3.3
Ethnicity	White	196	56.6	166	90.2
	Black/Black British	40	11.6	5	2.7
	Asian/Asian British	60	17.3	7	3.8
	Mixed	25	7.2	4	2.2
	Other Ethnic Group	25	7.2	2	1.1
Employment Status	Employed – Full time	N/A		102	55.4
	Employed – Part time or Zero Hours Contract			25	13.6
	Self-Employed			31	16.8
	Stay at Home Caregiver			6	3.3
	Unemployed			20	10.9
Profession	Manager			28	18.2
	Professional			76	49.4
	Technician or Associate Professional			12	7.8
	Clerical Support Worker			15	9.7
	Skilled Agricultural			12	7.8
	Craft or Related Trade Worker			1	0.6
	Plant or Machine Operator			5	3.2
	Elementary Occupation			1	0.6
	Armed Forces			2	1.3

#### 3.1.2 Materials

##### 3.1.2.1 Male rape myths

A revised 41-item version of the Male Rape Myth Acceptance Scale (MRMAS) from Study 1 was administered to participants. These items again assessed beliefs relating to Masculinity Sexuality, Pleasure, Effect, Context, and Perpetrators. Importantly, following analysis from Study 1, this scale now has two identified sub-scales – a 23 item Blame subscale, and an 18 item Minimisation/Exoneration subscale. Participants answered all items on a 7-point Likert scale between 1 (Strongly Disagree) and 7 (Strongly Agree). The reliability estimates for the 41 item model in this sample using Maximum Likelihood estimation with Pearson (Polychoric) correlations and a Promax rotation were Alpha = .97 (.97), Hierarchal omega = .79 (.74), Omega total = .97 (.98). The Cronbach's alpha and hierarchal omega demonstrates reliability for a general factor solution, however the discrepancy in the Hierarchal omega and Omega total coefficients suggests the variance in the 41 items is likely to be attributable to a multiple factor structure or specific item variances. Cronbach's alpha values for the study 1 version of the subscales 1 (Blame) and 2 (Minimisation/Exoneration) were .96 and .91 respectively.

An additional measure of male rape myths was also administered. Namely, the six items from Struckman-Johnson and Struckman-Johnson (1992) rape myth measure, which describe a man victimised by another man. Two items measured myths surrounding trauma (e.g., “Most men who are raped by a man do not need counselling after the incident”), two measured denial (e.g., “It is impossible for a man to rape a man), and two measured blame (e.g., “Most men who are raped by a man are somewhat to blame for not being more careful). This measure uses a 6-point Likert scale (1 = strongly disagree, 6 = strongly agree), with higher scores indicating more endorsement of these rape myths. The Cronbach's alpha in the present sample was .76 (.88), Hierarchal omega = .57 (.82) and Omega total = .87 (.93), suggesting acceptable unidimensional reliability when estimated using Polychoric correlations.

##### 3.1.2.2 Female rape myths

The Acceptance of Modern Myths About Sexual Aggression (AMMSA) scale (Gerger et al., 2007) provided a measure of female rape myth acceptance. Participants answered 30 questions, using a Likert scale ranging from ‘strongly disagree’ to ‘strongly agree’. Examples include “When it comes to sexual contacts, women expect men to take the lead” and “If a woman invites a man to her home for a cup of coffee after a night out this means that she wants to have sex”. This measure was selected as it overcomes many of the pitfalls associated with historical measures of rape myth acceptance (e.g. Rape Myth Acceptance Scale – RMAS; Burt, 1980; Attitudes Towards Rape Scale – ATR; Feild, 1978; Illinois Rape Myth Acceptance Scale – IRMAS; Payne et al., 1999), (for details see (Bohner et al., 2009; Gerger et al., 2007; Hinck and Thomas, 1999; Payne et al., 1999). The Cronbach's Alpha of the AMMSA in the present sample was .94 (.95), Hierarchal omega = .77 (.85) and Omega total = .95 (.96), in keeping with previous Alpha levels of .92 (Gerger et al., 2007).

##### 3.1.2.3 Homophobia

A modified 27-item version of the LGB-KASH (Worthington et al., 2005) was administered to measure attitudes toward LGB individuals. It contained the following five subscales: Hate (6 items), LGB Knowledge (5 items), LGB Civil Rights (4 items), Religious Conflict (7 items), and Internalised Affirmativeness (5 items). One LGB Civil Rights item relating to provision of healthcare for same-sex couples was removed, as, in the UK, healthcare is free to all at the point of delivery. Items relating to LGB knowledge were also modified to represent knowledge of UK LGB history. All items are rated on a Likert-type scale ranging from 1 (very uncharacteristic of me or my views) to 6 (very characteristic of me or my views). Higher scores on each subscale are indicative of higher levels of the construct measured by that subscale. Examples of items include the following: “I think marriage should be legal for same-sex couples” (LGB Civil Rights); “Feeling attracted to another person of the same sex would not make me uncomfortable” (Internalised Affirmativeness); “I am knowledgeable about the history and mission of the PFLAG organization” (LGB Knowledge); “I sometimes think about being violent toward LGB people” (Hate); and “I keep my religious views to myself in order to accept LGB people” (Religious Conflict). Worthington et al. (2005) reported evidence for the validity of the scale via findings of hypothesised relationships with social dominance orientation, sexual identity development, homophobia and biphobia, age, gender, and sexual orientation identity. They report internal consistencies from .73 to .94 for the various subscales in three separate studies. In this sample the Cronbach's alpha estimate for all items in the scale overall was .91 (.94), while the Hierarchal Omega coefficient was .52 (.64) and the Omega total was .93 (.96) suggesting a multivariate structure was appropriate as previously determined by Worthington et al. (2005). Alpha Scores varied for the five subscales: Hate (.82), LGB Knowledge (.85), LGB Civil Rights (.85), Religious Conflict (.78), and Internalised Affirmativeness (.35) subscales.

### 3.2 Results

Descriptive statistics, including item means, item distribution statistics and item-to-total correlations were produced. An exploratory factor analysis was also computed to ratify the presence of the two-factor solution identified in Study 1. The aim was to then use Confirmatory factor analyses to compare the general factor, two factor (Blame and Minimisation/Exoneration) and six factor solution (Masculinity, Sexuality, Effect, Context, Pleasure, Perpetrator) in the Study 2 data to see how each solution fits. Group differences were assessed through the computation of several t-tests. Correlations were also conducted between the MRMAS measures (in models displaying good fit) and other proximal constructs.

#### 3.2.1 Descriptive statistics

Item means, skewness statistics and item-total correlations for the revised MRMAS are shown in Table 4. Overall, item means were still towards the middle to lower part of the Likert scale, showing that data remained positively skewed (supported by the raw item skewness statistics). However, item-total correlations again demonstrated that most items correlated strongly to the total.

**Table 4:** Descriptive statistics for each MRMAS item – study 2.

Item	Code	Mean	SD	Item-Total Correlation	Skewness raw item scores	Skewness SQRT transformed	Skewness LG10 transformed
1. If a man is raped it does not mean he is weak	1M.C.A.R	2.28	1.85	.437	1.410	1.059	0.757
2. A man who is raped must have been behaving in a way that made him appear homosexual	1S.B.A	1.80	1.28	.755	1.603	1.261	1.006
3. Male rape is very rare, if it occurs at all	1C.T.C.	2.67	1.51	.568	0.600	0.242	-0.096
6. Male on male rape only happens to homosexual men	2S.C.C.	1.82	1.21	.774	1.438	1.111	0.858
7. A male victim who ejaculates during the incident has not been raped	1PL.B.C.	1.80	1.19	.685	1.549	1.151	0.863
8. Almost all male rape occurs in institutions such as prisons or the military	2C.T.C.	2.68	1.48	.547	0.589	0.208	-0.145
9. A homosexual man who rapes other men does so out of sexual desire	2P.B.C.	3.47	1.58	.418	0.057	-0.369	-0.786
10. Most men would be able to fight off a male sexual attacker	3M.C.C.	2.66	1.45	.653	0.588	0.224	-0.131
11. Rape is an accepted risk of a ‘homosexual lifestyle’	3S.B.A.	1.80	1.23	.743	1.518	1.193	0.939
14. Heterosexual men who commit rape do so to act upon secret homosexual desires	3P.C.C.	3.19	1.56	.478	0.163	-0.226	-0.579
15. In ‘real’ cases of male rape, there will be some evidence of physical resistance	4M.B.C.	3.03	1.72	.519	0.413	0.072	-0.256
16. Heterosexual men ‘cry rape’ to hide their homosexual activities	4S.B.A.	2.40	1.39	.681	0.631	0.329	0.065
17. Even if force is used to initiate sex, the victim's erection can be interpreted as pleasure	3PL.B.C.	2.03	1.35	.647	1.203	0.869	0.601
18. I would expect heterosexual victims of rape to be more traumatized than homosexual victims	1E.C.A.	2.32	1.61	.646	1.069	0.728	0.443
19. Coercive sexual practices between men (e.g., forced oral sex) form a legitimate part of group initiations such as those used in fraternities or sporting societies	4C.T.C.	2.16	1.36	.513	0.817	0.575	0.371
20. A man would not rape another man if he was sexually fulfilled elsewhere	4P.B.C.	2.11	1.40	.635	1.256	0.859	0.534
21. Just because a man is raped does not mean he is homosexual	5S.C.C.R	2.05	1.69	.492	1.604	1.289	1.007
22. Men should feel ashamed as a result of being raped	2E.C.A.	1.74	1.29	.678	1.852	1.462	1.181
23. Most cases of male rape include the use of a weapon	5C.T.C.	3.36	1.30	.345	-0.394	-0.762	-1.115
24. Male rape is only perpetrated by homosexual men	5P.C.C.	2.07	1.34	.731	1.145	0.808	0.525
25. A homosexual man who has been raped probably enjoyed the experience to some extent	4PL.C.A.	1.71	1.19	.768	1.173	1.389	1.132
26. A man is more responsible for his own rape if he frequents a known homosexual area or establishment	6C.B.A.	1.77	1.24	.832	1.592	1.284	1.037
27. Only men who are big and strong are able to rape other men	6P.C.C.	1.95	1.27	.791	1.316	0.962	0.661
28. I would find it difficult to consider a man a ‘real man’ if he said he had been raped	5M.C.A.	1.76	1.25	.806	1.694	1.351	1.091
29. If a man has already had consensual sex with other men, I would not believe his claims of rape	6S.B.A.	1.73	1.14	.830	1.637	1.261	0.974
30. A man who is raped is not as traumatized by the experience as a woman	3E.C.A.	1.84	1.31	.752	1.595	1.228	0.953
31. If a man is drunk or taking drugs he is accepting rape as a possible risk	6C.B.A	1.84	1.26	.685	1.506	1.162	0.882
32. Men who commit rape are naturally more aggressive in their day to day lives	7P.C.C.	3.30	1.60	.272	0.160	-0.231	-0.611
33. It is acceptable for a ‘real man’ to show fear during a sexual attack by another man	6M.B.A.R	2.38	1.87	.414	1.208	0.917	0.642
34. A man who claims to have been raped probably just changed his mind after initially consenting to sex	7S.B.A.	1.87	1.16	.801	1.244	0.916	0.652
35. A male victim's reaction to rape is more likely to be practical than emotional (e.g., obtaining a HIV test rather than seeking support)	4E.B.C.	3.14	1.70	.285	0.247	-0.092	-0.400
36. A male victim of rape must have behaved in a way that invited the assault	7C.B.A.	1.74	1.13	.826	1.625	1.241	0.946
37. A heterosexual man who had been raped would still be desirable to women	7M.C.A.R.	2.75	1.78	.519	0.842	0.485	0.130
38. Male rape is a homosexual act	8S.C.C.	2.80	1.76	.528	0.709	0.336	0.004
39. If a man has been raped he should be able to cope on his own	5E.C.A.	1.79	1.24	.757	1.652	1.261	0.965
40. I would find it difficult to believe a man had been raped if he had previously consented to sex with the same man	8C.B.A.R.	2.01	1.35	.769	1.343	0.963	0.653
41. Regardless of how they identify themselves, I believe that men who rape other men are homosexual	8P.C.A	2.69	1.71	.588	0.794	0.408	0.073
42. ‘Real men’ cannot be raped	8M.C.A.	1.61	1.16	.773	2.149	1.721	1.403
43. I would expect a man to be ‘matter of fact’ and in control of his emotions when reporting a rape	6E.B.A.	1.98	1.35	.745	1.377	1.010	0.708
44. A man who has been raped did not set sexual limits understood by the perpetrator	9C.B.C.	2.02	1.36	.681	1.366	0.950	0.636
45. Male victims of rape have very little emotional trauma to cope with	7E.C.A.	1.63	1.16	.753	1.997	1.617	1.335

Note: Skewness SE = 0.106. Code is comprised of a) a number denoting the position of that item within each theme, b) a letter denoting the theme (M = Masculinity, S = Sexuality, PL = Pleasure, E = Effect, C = Context, P = Perpetrator), c) whether the item is Behavioral (B), Characterological (C) or Typological (T), and d) whether the item is Affective (A) or Cognitive (C). If the item also has an R at the end of its code, this means the question is reverse scored.

#### 3.2.2 Exploratory factor analysis

All of the parameters identified in Study 1 (e.g., coefficients values, Kaiser-Meyer-Oklin values etc.) were met in this sample also. Using the Scree plot, Parallel Analysis, inspecting cumulative variance explained, and running the VSS test suggested a two-factor extraction was appropriate. The Velicer's MAP test suggested a four factor structure, however upon review of the indices the third and fourth factors added little to the model, so we proceeded with the two factor extraction. Exploratory factor analysis was again conducted using the Principal Axis Factoring (PAF) technique with a promax (oblique) rotation then applied due to the degree of positive skewness observed in item responses. The two factor model explained 50.75% of the variance. A suppression threshold of .42 was chosen to ensure secure factor loadings onto a factor. Factor loadings for each item are presented in Table 5.

**Table 5:** Exploratory factor loadings using the Principal Axis Factoring technique for each MRMAS item – study 2

Item	Factor 1 – Blame	Factor 2 – Minimisation/Exoneration
42. ‘Real men’ cannot be raped	.928	
45. Male victims of rape have very little emotional trauma to cope with	.912	
21. Just because a man is raped does not mean he is homosexual	.878	-.409
2. A man who is raped must have been behaving in a way that made him appear homosexual	.874	
33. It is acceptable for a ‘real man’ to show fear during a sexual attack by another man	.833	-.443
36. A male victim of rape must have behaved in a way that invited the assault	.818	
6. Male on male rape only happens to homosexual men	.807	
37. A heterosexual man who had been raped would still be desirable to women	.785	
26. A man is more responsible for his own rape if he frequents a known homosexual area or establishment	.768	
39. If a man has been raped he should be able to cope on his own	.768	
29. If a man has already had consensual sex with other men, I would not believe his claims of rape	.767	
28. I would find it difficult to consider a man a ‘real man’ if he said he had been raped	.746	
25. A homosexual man who has been raped probably enjoyed the experience to some extent	.743	
43. I would expect a man to be ‘matter of fact’ and in control of his emotions when reporting a rape	.728	
22. Men should feel ashamed as a result of being raped	.687	
11. Rape is an accepted risk of a ‘homosexual lifestyle’	.672	
34. A man who claims to have been raped probably just changed his mind after initially consenting to sex	.657	
30. A man who is raped is not as traumatized by the experience as a woman	.665	
27. Only men who are big and strong are able to rape other men	.598	
**44. A man who has been raped did not set sexual limits understood by the perpetrator**	.590	
40. I would find it difficult to believe a man had been raped if he had previously consented to sex with the same man	.546	
1. If a man is raped it does not mean he is weak	.543	
7. A male victim who ejaculates during the incident has not been raped	.529	
31. If a man is drunk or taking drugs he is accepting rape as a possible risk	.511	
24. Male rape is only perpetrated by homosexual men	.416	
14. Heterosexual men who commit rape do so to act upon secret homosexual desires		.801
9. A homosexual man who rapes other men does so out of sexual desire		.768
15. In ‘real’ cases of male rape, there will be some evidence of physical resistance		.749
41. Regardless of how they identify themselves, I believe that men who rape other men are homosexual		.681
32. Men who commit rape are naturally more aggressive in their day to day lives		.665
23. Most cases of male rape include the use of a weapon		.595
16. Heterosexual men ‘cry rape’ to hide their homosexual activities		.586
20. A man would not rape another man if he was sexually fulfilled elsewhere		.568
38. Male rape is a homosexual act		.550
18. I would expect heterosexual victims of rape to be more traumatized than homosexual victims		.517
10. Most men would be able to fight off a male sexual attacker		.480
17. Even if force is used to initiate sex, the victim's erection can be interpreted as pleasure		.444
8. Almost all male rape occurs in institutions such as prisons or the military		.423
*3. Male rape is very rare, if it occurs at all*		
*19. Coercive sexual practices between men (e.g., forced oral sex) form a legitimate part of group initiations such as those used in fraternities or sporting societies*		
*35. A male victim's reaction to rape is more likely to be practical than emotional (e.g., obtaining a HIV test rather than seeking support)*		

Note _a_Items shown in bold loaded onto different factors in Study 2 than in Study 1. _b_Italicised items did not load on either factor at .42, and were thus eliminated from the scale and further analysis.

A two-factor solution demonstrated near simple structure with most items loading onto one of the two factors. All items (bar two, shown in bold) loaded on the same factors as in Study 1, further supporting the selection of a two-factor solution; victim blaming versus exonerating perpetrator/minimisation. Two items did not load onto either factor in this sample (items 3 and 19), while another two did not load at .42 (items 24 and 35). These items were identified for elimination. As the two factor structure has been maintained through a second PAF analysis, all of the 41 item scores were transformed towards greater normality of distribution in preparation for running the CFA models. Square root and Log 10 transformations were conducted separately on the raw item scores due to the trend towards positive skew in the raw item data. The skewness statistics suggest that the Log 10 transformation was more effective at normalising the distribution of the data (see Table 4). When correlations were performed between the raw item scores and the two different normalised transformations of the same item, the raw item scores were found to be correlated at .99 with the square root transformed scores, and at .97 with the Log 10 transformed scores. The same two factor structure was maintained in the transformed for normality data (both the Square root and Log 10 analyses separately), although item 24 maintained a loading above .42 in both of these transformations. Therefore items 3, 19 and 35 only were eliminated from the scale and subsequent analyses, as the raw item loading of item 24 was .416. This leaves a two factor solution containing 38 items overall (25 Blame, 13 Minimisation/Exoneration). The reliability estimates for the raw 38 item model in this sample using Maximum Likelihood estimation with Pearson (Polychoric) correlations and a Promax rotation were Alpha = .97 (.97), Hierarchal omega = .83 (.72), Omega total = .97 (.98). Cronbach's Alpha analyses showed factor 1 (Blame) and factor 2 (Minimisation/Exoneration) displayed excellent or close to excellent reliability (.96 and .89 respectively).

#### 3.2.3 Confirmatory factor analysis

Confirmatory factor analyses were conducted using Robust maximum likelihood estimation (MLM variant), which is available through the *Lavaan* package in R. Robust maximum likelihood estimation was used, as this allows for some violation in the normal distribution assumption when compared to the standard version (Schmidt, 2011). Comparisons will be performed in the model fit across the raw item scores, and the two sets of normalised item scores separately. The intention of using CFA is to compare the fit of the general factor, two factor (Blame and Minimisation/Exoneration) and six factor solutions (Masculinity, Sexuality, Effect, Context, Pleasure, Perpetrator) in the Study 2 data to see how well each solution fits.

In each of the models the error co-variances were set to be estimated between the items falling within each of the six type areas (Masculinity, Sexuality, Effect, Context, Pleasure, Perpetrator) as there is likely to be commonly shared item variance in the measurement of the items tapping into each of the six previously theory-determined areas that were used to inspire the creation of the items. Several different indices of model fit were estimated, including the chi square/df ratio (<3 = good), Comparative fit index (CFI, >.95 = good fit, >.90 = acceptable fit), Tucker-Lewis fit index (TFI, >.95 = good fit, >.90 = acceptable fit), Root mean square error of approximation (RMSEA, <.06 = good fit, <.08 = acceptable fit) and the standardised root mean square residual (SRMR, <.08 = good fit). These guidelines of model fit are based on reviewing literature debating fit indices published by Hu and Bentler (1998), Marsh, Hau and Wen (2004), Russell (2002), and Scmitt (2011). Hu and Bentler (1998) recommendations (“good fit”, outlined above) are the standard that analyses aim to adhere to, Marsh et al., (2004) highlight that in models with a larger number of variables/items (such as questionnaires) reaching these model fit criteria become challenging to meet, even at the “acceptable” levels outlined above (Russell, 2002, also briefly addresses this). The use of data that is non-normally distributed for theoretically valid reasons in its raw format (as we have in this study) further increases this challenge.

Table 6 provides a summary of the fit indices for the general factor and two factor solutions. Unfortunately, the six factor solution did not converge. Table 6 also highlights how many of the fit indices have met at least an acceptable level of fit in a particular model based on the guidelines above (last column). Note that due to the sample size collected the chi square/df ratio and SRMR may be displaying a bias towards better fit. This is why greater credence is being placed on a combination of two indices out of CFI/TLI and RMSEA meeting (at least) acceptable fit conditions. The two factor models consistently met this criteria, regardless of raw or normalised score transformation, with the two factor structure providing a better fit to the data than the general factor model. Although under conditions of normalised transformation of scores (Square root or Log 10 transformation) the general factor model starts to demonstrate acceptable levels of fit across two of the indices outlined above. The Log 10 transformed versions of the scores demonstrate a better degree of model fit to the data than both the square root transformed scores, and the raw scores, with the Two factor model estimated using Log 10 transformed scores meeting good levels of fit.

**Table 6:** A summary of the fit indices of the confirmatory factor analysis models run using the 38 items identified across both studies.

Model	Robust Chi square	Robust df	Robust Chi square/df	CFI(Robust)	TLI (Robust)	RMSEA (Robust)	SRMR	Model fit criteria suggesting at least acceptable fit
Baseline model, raw scores	10332.918	703	14.70					N/A
General factor model, raw scores	1597.99	556	2.87	.89 (.89)	.86 (.86)	.060 (.073)	.062	Chi square/df, RMSEA, SRMR
Two factor model, raw scores	1395.65	555	2.51	.91 (.91)	.89 (.89)	.054 (.065)	.057	Chi square/df, acceptable CFI, good RMSEA (standard), SRMR
Baseline model, SQRT scores	13478.129	703	19.17					N/A
General factor model, SQRT scores	1633.71	556	2.94	.92 (.90)	.89 (.88)	.061 (.071)	.060	Chi square/df, CFI, RMSEA, SRMR
Two factor model, SQRT scores	1411.70	555	2.54	.93 (.92)	.92 (.90)	.054 (.064)	.055	Chi square/df, CFI, TFI, good RMSEA (standard), SRMR
Baseline model, LG10 scores	16533.831	703	2.52					N/A
General factor model, LG10 scores	1610.63	556	2.90	.93 (.91)	.92 (.89)	.060 (.069)	.057	Chi square/df, CFI, TFI(standard), RMSEA, SRMR
Two factor model, LG10 scores	1376.61	555	2.48	.95 (.93)	.93 (.92)	.053 (.061)	.052	Chi square/df, good CFI (standard), TFI, good RMSEA (standard), SRMR

Note: Error variances were set to be correlated between the items falling within each of the six item type criteria (Masculinity, Sexuality, Pleasure, Effect, Context, Perpetrator). Abbreviations: SQRT = Square root transformed, LG10 = Log 10 transformed scores, df = degrees of freedom, CFI = Comparative fit index, TLI = Tucker-Lewis fit index, RMSEA = Root Mean Square Error of Approximation, SRMR = Standardized Root Mean Square Residual.

Following on from the confirmatory factor analyses supporting the existence of the general factor (under normal transformations) and the two factor solution, descriptive statistics were run on the total scores calculated for all measures in the study (presented in Table 7).

**Table 7:** Descriptive statistics for all scales – study 2.

		N	Range	Minimum	Maximum	Mean	SD	Skewness	Kurtosis
Male Rape Myth Acceptance Scale (MRMAS) – 38 items	Men	204	4.50	1.03	5.53	2.63	1.01	0.559	-0.352
	Women	324	4.39	1.00	5.39	1.94	0.74	1.263	1.614
	Students	346	4.53	1.00	5.53	2.40	0.99	0.780	-0.093
	General Pop	182	2.45	1.00	3.45	1.83	0.61	0.807	-0.174
	Total	528	4.53	1.00	5.53	2.20	0.92	1.013	0.559
Male Rape Myth Acceptance Scale (MRMAS) – 25 items Blame subscale	Men	204	4.76	1.00	5.76	2.36	1.13	0.701	-0.456
	Women	324	4.32	1.00	5.32	1.63	0.77	1.673	2.618
	Students	346	4.76	1.00	5.76	2.14	1.09	0.888	-0.168
	General Pop	182	2.20	1.00	3.20	1.47	0.52	1.326	1.090
	Total	528	4.76	1.00	5.76	1.91	0.99	1.264	0.887
Male Rape Myth Acceptance Scale (MRMAS) – 13 items Minimisation/Exoneration subscale	Men	204	5.23	1.08	6.31	3.15	1.03	0.067	-0.532
	Women	324	4.54	1.00	5.54	2.53	0.91	0.418	-0.314
	Students	346	5.31	1.00	6.31	2.91	1.02	0.258	-0.442
	General Pop	182	4.15	1.00	5.15	2.51	0.92	0.417	-0.525
	Total	528	5.31	1.00	6.31	2.77	1.01	0.330	-0.453
Acceptance of Modern Myths of Sexual Aggression (AMMSA) – 30 items	Men	197	5.27	1.10	6.37	3.22	1.01	0.236	-0.035
	Women	315	4.07	1.00	5.07	2.57	0.91	0.330	-0.875
	Students	336	5.37	1.00	6.37	2.91	0.99	0.190	-0.409
	General Pop	176	4.87	1.17	6.03	2.65	0.99	0.671	0.047
	Total	512	5.37	1.00	6.37	2.82	0.99	0.344	-0.367
Lesbian, Gay, and Bisexual Knowledge and Attitudes Scale for Heterosexuals (LGB-KASH) – 27 items	Men	192	3.52	1.19	4.70	2.96	0.74	-0.320	-0.418
	Women	318	3.93	1.22	5.15	2.43	0.74	0.582	-0.372
	Students	337	3.96	1.19	5.15	2.80	0.77	-0.040	-0.868
	General Pop	173	344	1.19	4.63	2.29	0.68	0.779	0.456
	Total	510	3.96	1.19	5.15	2.63	0.78	0.226	-0.816
Struckman-Johnson & Struckman-Johnson Rape Myth Scale (SJ-SJ RMS) – 6 items	Men	200	4.50	1.00	5.50	1.99	0.96	0.690	-0.463
	Women	318	3.83	1.00	4.83	1.57	0.74	1.389	1.626
	Students	341	4.50	1.00	5.50	1.91	0.94	0.774	-0.284
	General Pop	177	2.33	1.00	3.33	1.38	0.52	1.488	2.12
	Total	518	4.50	1.00	5.50	1.73	0.86	1.107	0.518

For the MRMAS measures these displayed low mean scores as would be expected, although there was a general trend towards the Minimisation/Exoneration subscale means displaying a slightly higher average when compared to the Blame subscale. As this may be an artifact of the number of items in each subscale, to test whether there were any significant within participants difference in scores on these two subscales in each of the outlined demographic categorisations in Table 7, Z score versions of these subscale variables were created in SPSS. This was designed to place each of the subscale scores in the same unit of measurement (using the total sample mean and SD of each subscale in the calculation of that Z score variable). These were then compared using a selection of Paired sample t-tests where the file was split by one of the demographic grouping variables (Sex or Student/General population). Neither of the Sex categories (Male or Female) displayed significant within participant differences in Blame and Minimisation/Exoneration Z scores. However, the Student group and the General population group did. The Student group displayed significantly higher levels of Blame than Minimisation/Exoneration (Blame mean Z = 0.23, Minimisation/Exoneration mean Z = 0.14, *t* (345) = 2.054, *p* = .041), while the general population group displayed significantly higher levels of Minimisation/Exoneration than Blame (Blame mean Z = -0.44, Minimisation/Exoneration mean Z = -0.26, *t* (181) = -3.607, *p* < .001). It is worth noting that the Student group displayed higher levels of both Blame and Minimisation/Exoneration characteristics than the General population group (note the mean across all Z scores is 0, this direction is also supported by Table 7).

### 3.3 Relationships between variables

Additionally, in order to assess the construct validity of the MRMAS scales, it was administered alongside a number of conceptually proximal variables (as described above under materials; see Table 8).

**Table 8:** Construct validity Pearson correlations.

	1	2	3	4	5	6	7	8	9	10	11	12
1. MRMAS-Total	-											
2. MRMAS-Blame	.96∗∗∗	-										
3. MRMAS-Exon	.86∗∗∗	.68∗∗∗	-									
4. Age	-.21∗∗∗	-.22∗∗∗	-.14∗∗	-								
5. Coded sexuality	-.22∗∗∗	-.19∗∗∗	-.22∗∗∗	-.01	-							
6. LGB-KASH hate	.53∗∗∗	.54∗∗∗	.40∗∗∗	-.18∗∗∗	-.16∗∗∗	-						
7. LGB-KASH knowledge	.56∗∗∗	.52∗∗∗	.52∗∗∗	-.14∗∗	-.40∗∗∗	.25∗∗∗	-					
8. LGB-KASH Rights	.67∗∗∗	.71∗∗∗	.46∗∗∗	-.17∗∗∗	-.20∗∗∗	.49∗∗∗	.52∗∗∗	-				
9. LGB-KASH religious	.24∗∗∗	.21∗∗∗	.25∗∗∗	-.10∗	-.13∗∗	.44∗∗∗	.15∗∗	.13∗∗	-			
10. LGB-KASH IA	.50∗∗∗	.47∗∗∗	.47∗∗∗	-.09	-.43∗∗∗	.29∗∗∗	.62∗∗∗	.53∗∗∗	.24∗∗∗	-		
11. AMMSA	.55∗∗∗	.47∗∗∗	.59∗∗∗	-.07	-.15∗∗	.48∗∗∗	.32∗∗∗	.27∗∗∗	.33∗∗∗	.35∗∗∗	-	
12. SJ-SJ MRS	.69∗∗∗	.75∗∗∗	.43∗∗∗	-.20∗∗∗	-.12∗∗	.53∗∗∗	.38∗∗∗	.54∗∗∗	.25∗∗∗	.33∗∗∗	.41∗∗∗	-

*Note:* ∗p < .05. ∗∗p < .01. ∗∗∗p < .001.

Categorical variables included for complete output only.

Highest correlations were, as anticipated, present for those variables most proximal, and sharing greatest conceptual overlap. In order of strength of relationships, a significant positive correlation was noted between the MRMAS total and Struckman-Johnson and Struckman-Johnson Male Rape Scale (1992), *r* (514) = .69, *p* < .001; The MRMAS and the *rights* sub scale of the LGB-KASH inventory, *r* (506) = .67, *p* < .001; with slightly weaker relationships to the LGB-KASH *knowledge*, *r* (506) = .56, *p* < .001; The AMMSA measure of modern myths directed towards male on female rape (Gerger et al., 2007), *r* (508) = .55, *p* < .001; The LGB-KASH *hate* subscale, *r* (506) = .53, *p* < .001; and The LGB-KASH *internalised affirmativeness* subscale, *r* (506) = .50, *p* < .001, were also all significantly, positively correlated. The MRMAS subscales both displayed trends in a similar direction, although there was some divergence in the correlations to LGB-KASH *rights*, with Blame displaying a much stronger correlation, *r* (506) = .71, *p* < .001, than Minimisation/Exoneration, *r* (506) = .46, *p* < .001. The same pattern was displayed with correlations to the Struckman-Johnson and Struckman-Johnson Male Rape Scale, with Blame displaying a much stronger correlation, *r* (514) = .75, *p* < .001, than Minimisation/Exoneration, *r* (514) = .43, *p* < .001. The opposing direction was found for the correlations to the AMMSA with Blame displaying a slightly weaker correlation, *r* (508) = .47, *p* < .001, than Minimisation/Exoneration, *r* (508) = .59, *p* < .001.

## 4 Discussion

This study introduces a new measure of male rape myth acceptance, appropriately named the Male Rape Myth Acceptance Scale (MRMAS). As such, this study provides the first highly reliable, peer reviewed, comprehensive measure of male rapes myths specifically. It is argued that development of such a scale will allow for future measurement of male rape myths, across a variety of populations, institutions and settings, as well as allowing for the correlation of such attitudes with judgments towards male victims and reporting. As with ‘traditional’ rape myths, the opportunity to conduct research of this nature on male victims should allow for the development of training, educational programs and interventions targeting negative beliefs about the crime of male-on-male rape, in attempts to improve the experiences of victim-survivors upon disclosure.

### 4.1 The Male Rape Myth Acceptance Scale (MRMAS)

As mentioned above, the MRMAS achieved excellent levels of reliability across various methods as assessment. Favorable reliability scores were complimented by high levels of correlation with proximal measures (in support of previous findings by Walfield, 2018), including measures of traditional rape myths (i.e., the AMMSA), previous measures of male rape myths (i.e., the Struckman-Johnson and Struckman-Johnson scale), and other attitudes (i.e., homophobia), thus suggesting excellent construct validity. In this sense, it can be confidently claimed that the MRMAS provides a robust measure of myths relating to male-on-male rape, and that this measure it improves upon previous scales which have not reported strong reliability values (such as Struckman-Johnson and Struckman-Johnson, 1992 scale). It is also important to note that the MRMAS assesses agreement with a greater variety of myths than previous measures (including Melanson’s 1999 scale), with items assessing masculinity, sexuality, pleasure, perpetrators, context and effect. This, along with a greater number of items overall, also allowed for a more comprehensive assessment of latent structure. A two-factor structure was revealed, suggesting a grouping of items which blame the victim or suggest that only certain groups of men are raped, and those which exonerate the perpetrator or minimise the incident. Indeed, Omega totals demonstrate that this multiple factor was most appropriate (though reliability for the scale overall was still excellent, as measured by Cronbach's Alpha and Hierarchial omega). This provides valuable information as to the possible function of said myths, and how participants may be using different subsets of myth to support their reasoning and responses to victimisation (similarly to processes identified in research using traditional rape myths; see Bohner et al., 2009). Importantly, this moves discussions beyond the thematically-driven six factor structure proposed earlier in this piece; suggesting instead that myths are grouped by the broader latent structures around blame versus minimisation/exoneration for the victim and perpetrator respectively.

### 4.2 Implications

As detailed above, the MRMAS appears to provide an extensive, robust measure of male rape myth acceptance. In the introduction of this paper, it was argued that producing such a scale would allow for the proliferation of literature on male rape; an endeavor that is, arguably, long overdue (M. Davies and Rogers, 2006). In this sense, this study represents a ‘call to arms’ to those interested in both attitudes and responses towards incidences of rape and those involved, to develop research utilizing this scale, and thus greater insight into the experiences and responses to male victimization.

In determining where to begin, the literature exploring attitudes towards incidences involving female victims provides a useful framework. Specifically, examining the beliefs, judgments and actions of those in specialist populations (e.g., those within the criminal justice system, counsellors etc.) provides an important and highly impactful route to improving the experiences of male victims. For example, the existence of robust ‘traditional’ rape myth acceptance scales has allowed for the measurement of rape myth acceptance within police officers (Murphy and Hine, 2019), the assessment of their impact on police judgments (Hine & Murphy, 2017, 2019), and the relationship between rape myths and case processing (Hine et al., 2021; Hohl and Stanko, 2015; Murphy et al., 2021). However at present, whilst it has been argued that male rape myths are held by police officers, and are influential in their case decision making and approach (Javaid, 2016; Rumney, 2008), these observations are anecdotal. This new measure thus provides a previously unavailable avenue to more rigorous, empirical assessment in this and other areas.

Engagement in such areas of research would allow for the development of informed and targeted training and educational programs on male rape myths, and their impact on responses to victimization (both by the victim themselves, and those around them). Indeed, traditional rape myth acceptance scales are routinely used to evaluate the success of police training programs on rape investigation (see (Lonsway, Welch, & Fitgerald, 2001) for example). As such, this new measure not only provides an empirical framework upon which to construct training programs specific to male rape, but to evaluate them also. It can further be argued that, alongside these specific research avenues, simply raising awareness of the existence and nature of male rape myths is important in and of itself. Particularly important is to incorporate relationships between attitudes into training programs; in the case of male rape myths, this would mean acknowledging how homophobic attitudes towards sexual minority men underpin these beliefs and constructing programs accordingly. In short, by successfully chronicling and measuring both broad male rape myth themes, and specific male rape myth examples, the validation of this measure provides a clear catalogue of extant male rape myths for specialist populations and the public at large.

### 4.3 Limitations

There are three principal limitations with the current study. First, as with any process of validation, some items were excluded during the process of analyses (for a variety of reasons: poor item-total correlations, item means, poor factor loadings, feedback from researchers regarding participant understanding etc). As a consequence, it should be noted that some themes lost more items than others. Specifically, whilst most ‘themes’ have between seven and nine items, pleasure myths are only represented by three items. However, the scale appears to be structured around a two-factor solution, and the number of items in each of these factors remains robust.

Second, as observed in the methods section of both studies, many of the items, and the scale overall, appears to suffer from floor effects. This is common in scales which measure emotive or sensitive topics (such as rape), and in scales which are among the first to measure a specific concept (similar issues were found for earlier traditional rape myth scales, such as the IRMAS; (Lonsway et al., 1999). Part of the issue may be the wording of questions, as, for this first scale, the wording was not veiled. Specifically, the word rape was used in several places, as were other words such as erection, which may make some participants uncomfortable, and lead them to answer in socially desirable ways. Whilst this was partially compensated for through the use of square root and LOG10 transformations, future research should perhaps look to develop more obviously valenced items, as occurred with the development of the AMMSA (Gerger et al., 2007).

Third and finally, the decision was taken in this study as part of scale development, based on the differential content and affect of male-female versus male-female myths, to only include items pertaining to male-on-male rape. This decision will allow for targeted and appropriate administration and application of this scale to cases involving men assaulted by other men (e.g., when examining the relationship of such attitudes to criminal cases), particularly in the UK (where the legislative definition defines rape as perpetrated by only men towards either men or women). This decision is also supported by the fact that the sample in this study was from the UK, and it may have been confusing for participants to answer items about ‘rape’ perpetrated by females. However, it is important to recognize that the measurement of distinct attitudes pertaining to the sexual assault of men by women is an important future research direction. Ergo, the generation of a more general ‘male sexual assault scale’, or the addition of a sub-scale to the scale generated in this study, can both be considered worthy and much needed endeavors.

## 5 Conclusion

It has been argued that research on male rape myths is around 20 years behind that on female rape myths (Davies and Rogers, 2006). One of the principal barriers to progress in this area is the absence of a reliable measure of male rape myth acceptance. The present study provides such a tool, in an attempt to facilitate increasing research in this area. It is hoped that the generation of scholarly work in this area, facilitated through the use of this scale, will result in improved understanding of male victimization, and subsequent reporting and coping behaviour. This, in turn, should enable more effective support of male victims, and improved victim experience, particularly within the criminal justice system.

## Declarations

### Author contribution statement

Benjamin A. Hine, Anthony D. Murphy: Conceived and designed the experiments; Performed the experiments; Analyzed and interpreted the data; Contributed reagents, materials, analysis tools or data; Wrote the paper.

Jamie S. Churchyard: Analyzed and interpreted the data; Wrote the paper.

### Funding statement

This research did not receive any specific grant from funding agencies in the public, commercial, or not-for-profit sectors.

### Data availability statement

The authors do not have permission to share data.

### Declaration of interests statement

The authors declare no conflict of interest.

### Additional information

No additional information is available for this paper.
